# Cortisol suppression impairs retrieval of emotional memory and changes its neural correlates immediately and four days post-treatment

**DOI:** 10.1038/s41386-026-02505-z

**Published:** 2026-07-23

**Authors:** Despina Antypa, Patrik Vuilleumier, Ulrike Rimmele

**Affiliations:** 1 Laboratory for Behavioural Neurology and Imaging of Condition, Department of Basic Neurosciences, University of Geneva, Geneva, 1211 Switzerland; 2 Swiss Center for Affective Sciences, University of Geneva, Geneva, 1202 Switzerland; 3 Emotion and Memory Laboratory, Faculty of Psychology and Education Sciences, University of Geneva, Geneva, 1205 Switzerland; 4 Laboratory of Cognitive Science, Department of Psychology, University of Western Macedonia, Florina, 53100 Greece; 5 Center for Interdisciplinary Study of Gerontology and Vulnerability (CIGEV), University of Geneva, Geneva, 1277 Switzerland

**Keywords:** Long-term memory, Stress and resilience

## Abstract

Emotional memories are hard to forget. Yet, past research showed that very high and very low levels of the stress hormone cortisol crucially affect the retrieval of emotional memories both immediately and in a long-lasting manner. With a double-blind, placebo-controlled, within-subject fMRI design involving humans, the present study investigated the acute and lasting effects of cortisol suppression on the neural memory representations for emotional events. The pharmacological suppression of the physiological morning cortisol rise impaired immediate memory retrieval and disrupted the involvement of key brain regions, but also persistently altered the neural memory representations for these emotional memories, tested four days later. These results indicate that cortisol suppression distorts the engagement of the emotional memory neural network and is linked to lasting changes in emotional memory representations, suggesting potential for future research on clinical strategies for attenuating distressing emotional memories.

## Introduction

Why is it so hard to forget negative emotional memories? From hurtful remarks to terrible losses and traumatic experiences, emotional memories mark the moment of their making and stand out years later. While experiencing emotional events, release of the stress hormones cortisol and noradrenaline promotes emotional memory formation, including encoding and consolidation, through concerted action within a complex brain network [1–4]. These emotional memories will later be easier to access and retrieve, and, for some of us, impossible to forget. Because memory formation is completed soon after the initial experience, memory retrieval has been proposed as a more suitable target for interventions and emotional memory modifications in health and disease [5, 6].

Past studies showed that either increasing or reducing the level of the stress hormone cortisol at retrieval can weaken the recall of already established emotional memories, indicating a curvilinear relation between the level of cortisol and emotional memory retrieval possibly due to the distinct effects of glucocorticoid receptor engagement [7–13]. Interestingly, the influence of pharmacologically reduced cortisol levels has been shown to persist beyond the acute impairment of emotional memory, up to a week later, when cortisol levels are back to normal [8, 9]. These findings suggest that pharmacological modulation of cortisol during retrieval may not only acutely impair emotional memory retrieval but may also induce longer-lasting post-retrieval alterations in emotional memory representations, highlighting the importance of assessing retrieval effects beyond the immediate testing session. Past studies and theoretical considerations have discussed plausible mechanisms underlying these long-term effects, including reconsolidation or memory integration processes, and/or disrupted retrieval effects [14–17].

Evidence from animal research suggests that cortisol suppression leads to emotional memory impairment because sufficient cortisol levels are required to engage the amygdala in emotional memory and its modulation of memory-related brain regions, such as the hippocampus (e.g. [18–20]). Imaging studies have confirmed that the retrieval of emotional memories in humans is associated with increased activity in the amygdala and hippocampus, as well as in the medial prefrontal cortex and entorhinal cortices [7, 8]. However, the neural correlates of the acute and long-lasting effects of cortisol suppression on emotional memory retrieval have not yet been studied in humans, and the mechanism underlying cortisol effects on emotional memory retrieval in humans remains unclear.

**Fig. 1:**
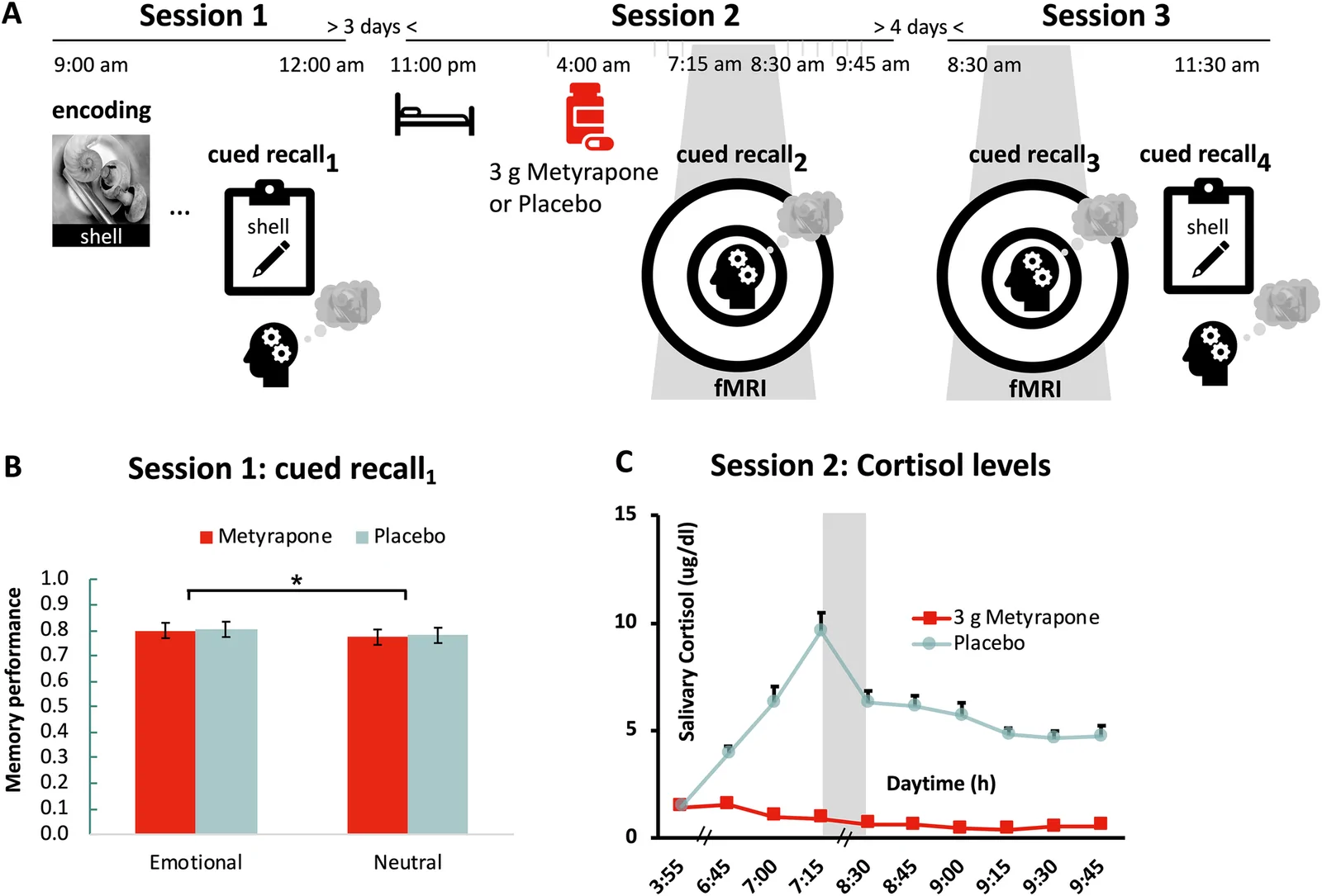
Experimental procedure cued recall after encoding at Session 1 and cortisol levels at Session 2. **A** Each participant was tested in three sessions under a metyrapone and a placebo condition, with the order of conditions counterbalanced across participants. In Session 1, participants learned 50 neutral and 50 emotional photo-word pairs, followed by an immediate cued recall to assess learning performance. In Session 2 (two days after encoding), participants slept in the lab, received treatment (3 g metyrapone/placebo) at 4:00 a.m., and performed a cued recall in the MRI scanner in the morning (between 7:15 and 9:00 a.m.; three days after encoding). In Session 3 (seven days after encoding), participants performed another cued recall in the scanner, followed by a final cued recall test outside the MRI to verify the memory probed during fMRI. **B** Cued recall performance during Session 1 did not differ between conditions later followed by metyrapone or placebo, with better recall of emotional images overall in both conditions. Error bars indicate SE and * *p* ≤ 0.05. **C** Cortisol levels: Mean ± SE salivary cortisol concentration at Session 2. The vertical gray bar indicates the time when fMRI data was collected. Baseline cortisol levels did not differ between conditions prior to treatment administration (at 3:55 a.m.), but metyrapone (red squares) suppressed cortisol levels for all the other time points.

For the present fMRI study, we adapted a previously validated paradigm [7, 8] to investigate acute and long-lasting effects of cortisol suppression on the neural circuits supporting memory for emotional events. At Session 1, participants learned word-photo associations with negative and neutral stimuli and were tested immediately afterwards with a self-paced cued recall task for which they had to provide written description of the recalled associated photos (Fig. 1A). At Session 2 (three days later), participants underwent fMRI while performing a cued recall task during the morning rise of cortisol under either placebo or pharmacologically suppressed cortisol levels due to the administration of metyrapone at 4 a.m. Four days after cortisol suppression (Session 3), participants underwent another cued recall during fMRI to assess the long-term effects of cortisol suppression on memory performance and related brain activity, and they were tested immediately afterwards with another self-paced cued recall task for which they had to provide written descriptions of the recalled associated photos. We expected that decreased cortisol levels would acutely impair the cued recall of emotional memories due to reduced amygdala engagement and interconnected brain regions involved in emotional memory retrieval, including the hippocampus and medial prefrontal cortex [5, 21]. We also predicted that cortisol suppression would disrupt emotional recall even later, when the drug is no longer effective, accompanied by persistent alterations in neural activity of this emotional memory retrieval network, potentially reflecting lasting changes in the neural memory representation of emotional events induced by cortisol suppression.

## Methods and materials

### Experimental design

Participants underwent testing in a randomized, placebo-controlled, double-blind, within-subject crossover design. After an adaptation night in the lab, each participant was tested under two conditions (metyrapone and placebo), with three sessions for each condition. The order of conditions was counterbalanced across subjects (Fig. 1A). To ensure that metyrapone was fully washed out before starting the second condition, an interval of at least ten days between Session 2 of the first condition and Session 1 of the second condition was arranged. Each condition included an encoding phase, immediately followed by a cued recall (Session 1; cued recall_1_). Three days after encoding, participants underwent a cued recall in the fMRI scanner either during their regular morning cortisol rise in the placebo condition or while their cortisol levels were suppressed in the metyrapone condition (Session 2; cued recall_2_). Session 2 took place during the early morning hours based on previous studies that reported that basal cortisol levels peak during that time of the day and can be suppressed by metyrapone [7, 22]. Seven days after the encoding session, i.e., four days after the cued recall under metyrapone or placebo from Session 2, participants underwent another cued recall in the fMRI scanner to investigate persistent memory changes and their neural representation, and another cued recall outside the scanner (Session 3; Fig. 1A; cued recall_3,_ cued recall_4_). During these sessions, participants engaged in cued recall, tasks, and questionnaires, described below and in *Psychological control variables* in Supplemental Information, and a reconsolidation task described elsewhere [23].

#### Session 1

In a morning session (9:00 a.m.–12:00 p.m.), participants learned pairs of photos and words, descriptive of the content of the paired photo, followed by an immediate cued recall test to assess encoding level. During the encoding task, photo-word pairs were presented in four blocks, with a maximum of three consecutive neutral or emotional pairs. Each pair was shown for 4 s, preceded by a white fixation cross for 2 s. Participants were instructed to form an association between the word and the image, as a cued memory task would follow.

Immediately after encoding, a self-paced cued recall task followed, during which cue words were presented in four blocks. Participants were asked to respond if they remembered the photo paired with each word (1 = yes, 2 = no). If they answered “yes,” they were asked to provide a sufficiently detailed written description of the photo for an external rater to identify it. In the cued recall, words were presented in four blocks according to the emotional content of the photos with which they were previously paired (two blocks paired with neutral photos and two with emotional); block order was counterbalanced across participants but remained consistent within the same participant across both conditions.

#### Session 2

Two days after encoding, participants spent the night in the laboratory (lights off at 11:00 p.m.) and were briefly awakened at 4:00 a.m. to orally receive either a placebo or metyrapone (3 g, HRA Pharma) along with a light snack (yogurt). Administration of this amount of metyrapone has been shown to effectively suppress the morning cortisol rise [7]. Later that morning, between 7:15 a.m. and 9:00 a.m. (three days after the encoding session), a cued recall test was conducted in the scanner. Participants were presented with words and asked to indicate if they remembered the photo paired with each word (1 = yes, 2 = no). If they recalled the photo, they were instructed to respond only after fully bringing the photo to mind as vividly as possible. Each word was displayed for 14 s, during which participants were asked to reconstruct and maintain the image in their mind’s eye (based on pilot tests, long cue duration allowed more reliable and sustained imagery of the retrieved photos). Cue words were preceded by a white fixation cross (jittered 1.5–2 s) and presented in four blocks, separated by 3-min rest intervals. Throughout Session 2, cortisol levels were assessed by collecting saliva samples at 3:55 a.m. (just before pill administration) and every 15 min from 6:45 a.m. to 9:45 a.m., except during the time participants spent in the scanner (see Fig. 1A).

#### Session 3

Seven days after encoding (Session 1) and four days after drug/placebo administration (Session 2), participants completed another cued recall task in the scanner between 8:30 and 10:30 a.m. (Session 3), with the same instructions as in Session 2. Immediately after scanning, participants provided a written description of their memories in response to each word through a self-paced cued recall task (as in Session 1).

### Participants

The study sample included 23 healthy participants (mean age 26.26 ± 4.61 years; mean body mass index 22.52 ± 2.3 kg/m²; four females and 19 males). All participants were non-smokers with a regular sleep-wake cycle, free from neurological, psychiatric, and endocrine disorders, and were not taking any medication during the study. They had no contraindications for metyrapone use or fMRI. The study was approved by the Cantonal Research Ethics Committee (Commission cantonale d’éthique de la recherche, CCER). All participants provided written informed consent and received financial compensation for taking part in the study. Some participants’ fMRI data are missing due to technical issues (Session 2: *n* = 2 males; Session 3: *n* = 2 males). Informed by a previous study examining the effects of metyrapone on memory, an a priori power analysis indicated that a sample size of 17 participants would be required to detect and effect of f = .482 with 95% power in a repeated-measures within-subject ANOVA (one group, α = .05) [7, 24]. Given the complexity of the study design (of eight sessions, incl. four fMRI scans and three overnights in the lab) the target sample size was increased to 23 participants to ensure adequate statistical power.

### Stimuli

During the encoding sessions, participants viewed 50 neutral photos and 50 emotional photos of negative valence, each paired with descriptive words. Two different sets of photo-word pairs were presented and counterbalanced between the two conditions across both sessions. All the photos were selected from the International Affective Picture Set [25]. The sets of emotional and neutral photos included a similar number of humans, animals, and inanimate objects, and were presented using E-Prime® software.

### Hormonal measures

In Session 2, Sarstedt Salivette tubes (Sarstedt, Rommelsdorf, Germany) were used to collect salivary cortisol samples at 3:55 a.m. (just before pill administration). Every 15 min from 6:45 a.m. to 9:45 a.m., except during the time participants were in the scanner (typically at 3:55, 6:45, 7:00, 7:15, 8:30, 8:45, 9:00, 9:15, 9:30, and 9:45 a.m.; see Fig. 1A). After collection, saliva samples were stored at -25 °C until they were sent for analysis to Dresden LABservice GmbH. Cortisol levels were determined using a competitive luminescence-enhanced enzyme immunoassay (IBL, Hamburg, Germany), with inter- and intra-assay coefficients of variation below 5%.

### Statistical analyses

#### Memory performance

Memory performance was analyzed using a 2 (metyrapone/placebo) x 2 (emotional/neutral) mixed-design analysis of variance (ANOVA) for each cued recall in Session 1, Session 2, and Session 3 (during and after scanning). Pairwise t-tests examined the disrupting effects of metyrapone on emotional memory, as reported in previous studies [8, 9]. In Sessions 1 and 3 (after scanning), memory performance was evaluated by an independent rater, unaware of the treatment, based on the written descriptions of the recalled photos, using pre-defined criteria from a previous study [26]. Greenhouse-Geisser corrections of degrees of freedom were applied when appropriate. The significance level was set to p ≤ .05.

#### Cortisol data analysis

Cortisol levels were analyzed using a linear mixed model (fitlme, MATLAB) that included fixed effects for treatment (placebo and metyrapone) and time (10 time points per condition), along with random effects for the subject factor. To achieve a more normal distribution of the residuals, cortisol levels were log-transformed (raw cortisol levels are shown in Fig. 1B).

### Imaging data acquisition and analysis

Functional images were acquired in a 3 T whole-body MRI scanner (Trio TIM, Siemens, Germany) with a 32-channel head coil, using a fast-multiplexed echo-planar imaging (EPI) sequence optimized for blood-oxygen-level-dependent contrast and high temporal resolution (see Supplemental Information for details). Preprocessing and analyses were performed with the use of Statistical Parametric Mapping software SPM8 (Welcome Trust Centre for Neuroimaging, London, UK) (see Supplemental Information for details).

After preprocessing, data from each participant was combined across runs and conditions to define 12 event types into a single general linear model (GLM) for each session (see Supplemental Information). Second-level group analyses were performed separately for the cued recall at Session 2 and the cued recall at Session 3. The main contrasts compared activations during the recall of emotional vs. neutral photos in the placebo and metyrapone conditions in Session 2 (under placebo or metyrapone), and on the later cued recall of Session 3 (4 days after placebo or metyrapone administration). We also tested the triple interaction of emotion (emotional/neutral) x memory (hits/misses), that is, the critical effect of remembered vs. forgotten photos in emotional compared to neutral context x drug condition (placebo/metyrapone) [E(HvsM)vsN(HvsM)_PLvsM], for Sessions 2 and 3. For this contrast, we excluded participants with fewer than six misses per condition (Session 2: *N* = 3, 2 females; Session 3: *N* = 4, 2 females). To define brain networks engaged in our memory paradigm across conditions, we examined main effects of emotional memory recall using FWE correction for multiple comparisons across the whole brain (*p* = .05 FWE, > 10 voxels). To test for predicted differences in memory networks between treatment conditions, we performed two planned interaction contrasts (emotion x treatment condition and emotion x treatment x recall success) using a more sensitive cluster-based threshold (*p* = .005 uncorrected, > 10 voxels), appropriate for subtle affective processes [27] and particularly those without precisely defined onsets [28] as long-term memory retrieval here. Although we focus on effects in memory-related brain regions which survived Small Volume Correction (SVC) and/or Family-Wise Error (FWE) correction, all activations are reported in the Supplemental Information.

## Results

### Session 1

#### Memory performance

Cued recall performance immediately after encoding did not differ between the two conditions, i.e. before receiving metyrapone or placebo in the subsequent session (all p > 0.793). Globally, participants recalled more emotional vs. neutral images [M_emo_ = 0.80, SE = 0.02 vs. M_neut_ = 0.78, SE = 0.02; main effect of emotion: F(1, 22) = 4.306, *p* = 0.050, η^2^ = .164; Fig. 1B]. These findings demonstrate similar baseline memory in both conditions and enhanced emotional memory in the present paradigm.

### Session 2

#### Memory performance

**Fig. 2:**
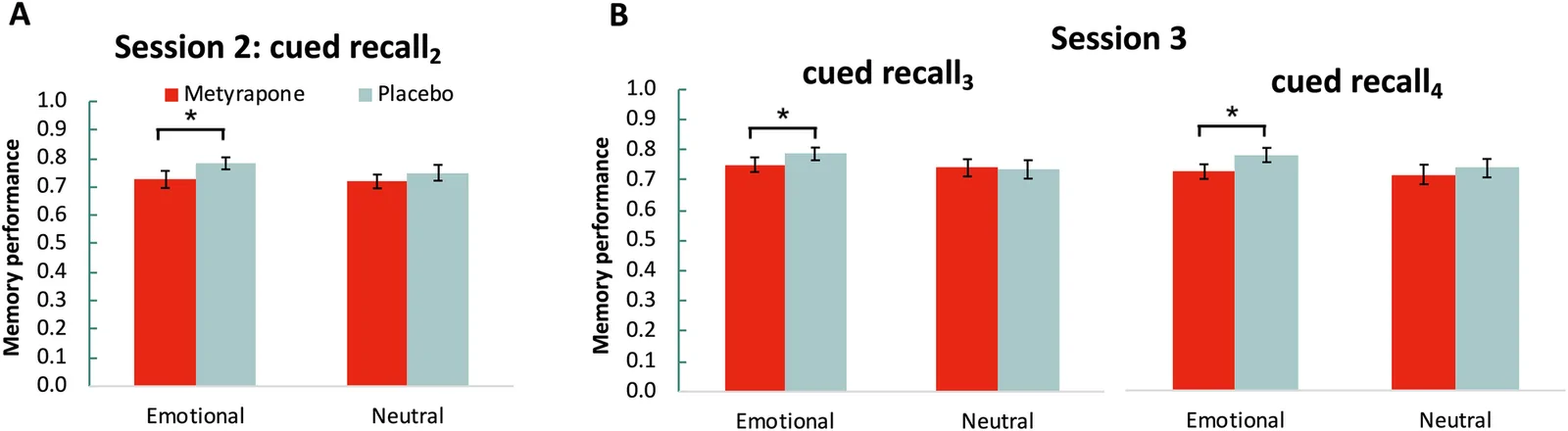
Memory performance at cued recall for emotional and neutral stimuli in the metyrapone and placebo conditions in Session 2 and Session 3. **A** In Session 2, metyrapone decreased memory performance for emotional and, to a lesser degree, neutral images during the cued recall task performed in the fMRI scanner. **B** In session 3, cued recall performance during the fMRI scan (cued recall_3_) and after the fMRI scan (cued recall_4_) revealed lower memory for emotional photos in the metyrapone vs. placebo condition, while memory for neutral photos did not differ between conditions. Error bars indicate SE and * *p* ≤ .05.

In the fMRI session performed three days after the encoding of image-word pairs, metyrapone decreased participants’ memory for previously learned photos (M_emo_ = 0.73, SE = 0.03; M_neut_ = 0.72, SE = 0.02) in comparison to placebo (M_emo_ = 0.78, SE = 0.02; M_neut_ = 0.75, SE = 0.03), when cued with the associated word [main effect of treatment: F(1,22) = 4.552, *p* = 0.044, η^2^ = 0.171]. Planned follow-up comparisons indicated mainly a decrease in memory for emotional photos in the metyrapone vs. placebo condition [t(22) = -1.917, *p* = 0.034, r = 0.38; one-tailed; Fig. 2A], while the difference in the same direction for neutral photos was not significant [t(22) = -1.463, *p* = 0.079, r = 0.29; one-tailed; Fig. 2B]. Although the numerical effect appeared larger for emotional than neutral photos, the absence of statistically significant treatment by emotion interaction does not support a definitive conclusion regarding differential effects of metyrapone on memory for emotional vs. neutral photos. Overall, these findings show that metyrapone administration acutely impairs memory retrieval, with more pronounced effects on emotional memory.

#### Cortisol levels

Treatment with metyrapone or placebo, time, and the interaction of treatment with time significantly predicted salivary cortisol levels at Session 2 [main effect of treatment: F(1, 411.94) = 1487.6, p < 0.001; main effect of time: F(10, 412.81) = 7.561, p < 0.001; treatment x time interaction: F(10, 411.58) = 22.286, p < 0.001]. Baseline cortisol levels at 3:55 am before treatment administration did not differ between the placebo and metyrapone conditions [placebo baseline: b = 0.063, t(434) = 1.011, *p* = 0.313; metyrapone baseline: b = -0.056, t(434) = -0.687, *p* = 0.492]; but there was a significant suppression of the natural morning cortisol rise: cortisol levels decreased for all time points after pill administration in the metyrapone vs. placebo condition [all *p* < 0.001; maximum difference at time-point 7:15 am, placebo: b = 0.857, t(434) = 10.914, *p* < 0.001; metyrapone: b = -1.145, t(434) = -10.209, *p* < 0.001; see Fig. 1C; Table S1].

#### Imaging results

**Fig. 3:**
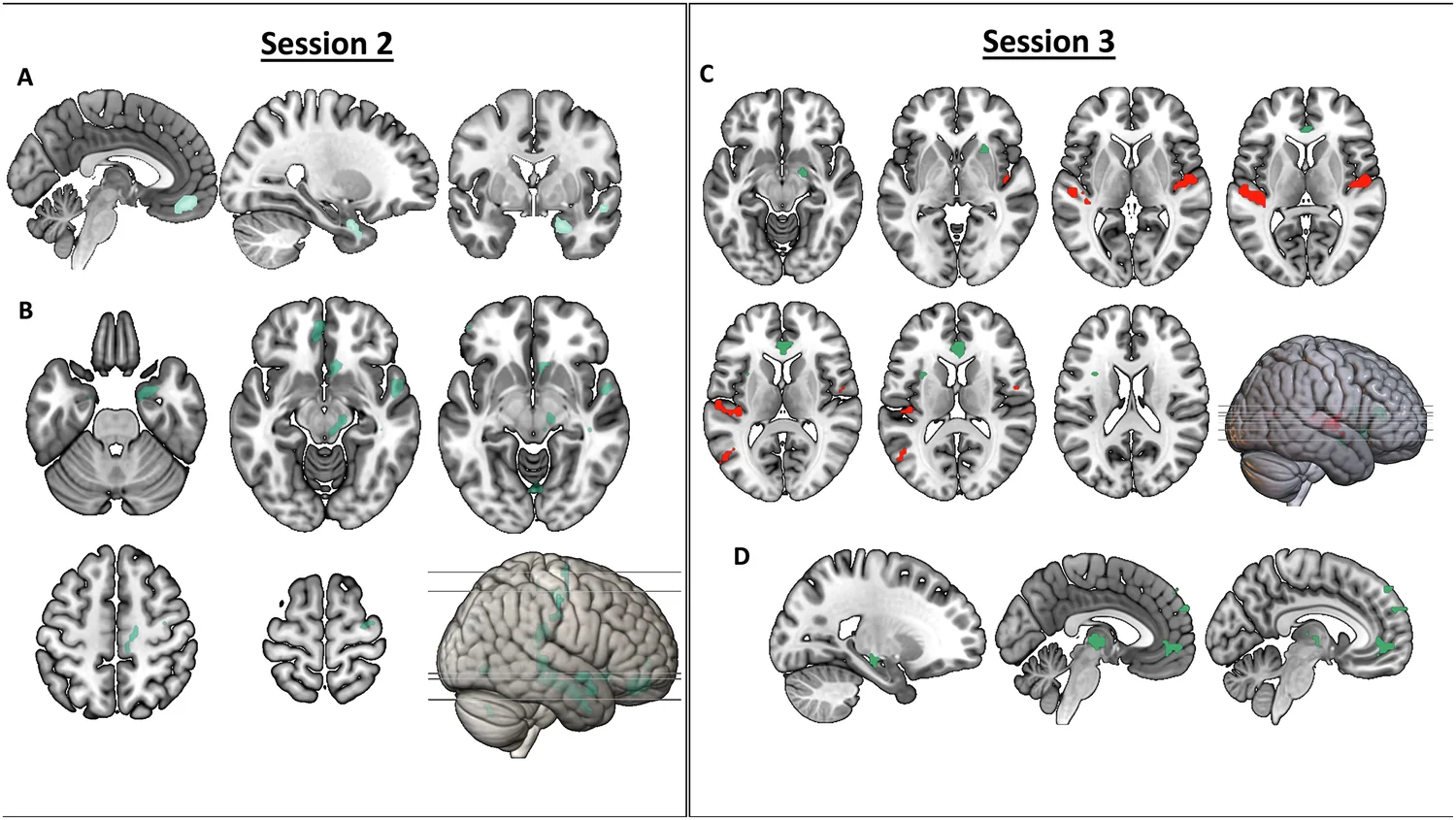
Sessions 2 & 3: Activations associated with the retrieval of emotional vs. neutral photos upon cue. **A** Session 2: Activations associated with the retrieval of emotional vs. neutral photos in the placebo condition (displayed at *p* = 0.05 FWE, > 10 voxels) include brain regions such as the left ventromedial prefrontal cortex, right amygdala, and right hippocampus. No activations are associated with retrieving emotional vs. neutral photos in the metyrapone condition (even with a more liberal threshold). **B** Session 2: Direct comparison of activations associated with the retrieval of emotional vs. neutral photos in the placebo vs. metyrapone conditions (*p* = 0.005, > 10 voxels). Activations of left ventromedial cortex, bilateral amygdala, and right hippocampus that survived SVC and FWE correction at cluster level. **C** Session 3: Activations associated with retrieval of emotional vs. neutral photos in the placebo condition (green) involved the right amygdala, right anterior insula, right putamen, and left anterior cingulate; the activation of amygdala survived SVC and FWE correction at cluster level. Activations associated with retrieval of emotional vs. neutral photos in the metyrapone condition (red) involved the right posterior insula and left transverse and middle temporal gyrus; no activation survived SVC and FWE correction at cluster level (*p* = 0.005, > 10 voxels). **D** Session 3: Triple interaction of recall x emotion x drug conditions. Differential activations for emotional (hits vs. misses) vs. neutral (hits vs. misses) in the placebo vs. metyrapone condition included the left hippocampus, left medial prefrontal cortex (including the left anterior cingulate), left temporal pole, and left superior frontal gyrus (*p* = 0.005, > 10 voxels). Activations of left hippocampus and medial prefrontal cortex survived SVC and FWE correction at cluster level.

Three days after encoding, we examined brain activity during cued recall under suppressed cortisol levels (after metyrapone) compared to cued recall under normal cortisol levels (after placebo). Using a whole-brain analysis and FWE correction for multiple comparisons across the whole brain, we compared recall of the different image types in each treatment condition. Under placebo, cued recall of emotional vs. neutral photos engaged several brain regions associated with episodic memory, including left ventromedial prefrontal cortex, right amygdala, and right hippocampus (*p* = 0.05 FWE, > 10 voxels; see Fig. 3A; Table 1A). In contrast, under suppressed cortisol levels, the same contrast for emotional vs. neutral recall showed no significant differential activity (even with a more sensitive threshold, *p* = 0.005 uncorrected, > 10 voxels). When directly testing for differences for cued recall of emotional vs. neutral photos in the placebo vs. metyrapone condition (interaction contrast), we confirmed a distinctive modulation of the same regions within left ventromedial cortex, bilateral amygdala, and right hippocampus, which survived SVC and FWE correction at cluster level (*p* = 0.005 uncorrected, > 10 voxels; Fig. 3B; Table S2). Furthermore, in the planned interaction contrast comparing emotional hits vs. misses relative to neutral hits vs. misses in the placebo vs. metyrapone condition, we found selective differences in only a few specific brain regions, including again right amygdala/hippocampus as well as parahippocampal regions (*p* = 0.005 uncorrected, > 10 voxels; see Table S3). These effects were anatomically selective, and no reliable effect (i.e., significant after similar thresholds) was observed outside these memory-related regions.

**Table 1:** Sessions 2 & 3: Emotional hits vs. neutral hits in placebo and metyrapone condition.

Region	Side	Number of Voxels	*Z* score	MNI coordinates (mm)
**Session 2**				
*Placebo condition*				
Parietal operculum	R	29	4.64	39 -28 22
Temporal pole	R	34	4.48	51 -1 -11
Amygdala/Hc/Ent/Phg	R	98	4.48	27 2 -29
	R		4.35	18 -4 -23
	R		4.35	15 -10 -14
Subcallosal area	R	17	4.46	9 20 -11
Temporal pole	R	23	4.45	42 -28 -5
Transverse temporal gyrus	L	22	4.44	-33 -31 16
Medial frontal cortex	L	63	4.38	-3 41 -17
Midcingulate cortex	R	13	4.34	15 -13 46
Middle frontal gyrus	L	10	4.22	-42 50 -2
Precuneus	L	10	4.10	-9 -49 40
Cerebellum	R/L	11	4.04	0 -73 -8
**Session 3**				
*Placebo condition*				
Anterior cingulate gyrus	L	42	2.84	0 29 13
**Amygdala**	R	12	2.80	21 -7 -11*
Insula (anterior)	L	11	2.77	-30 8 19
Putamen	R	13	2.77	24 17 -2
*Metyrapone condition*				
Insula (posterior)	R	61	3.11	51 -10 4
Transverse temporal gyrus	L	64	2.98	-54 -22 7
Middle temporal gyrus	L	14	2.95	-42 -25 10

Activations associated with the retrieval of emotional vs. neutral scenes, upon cue. At Session 2, the contrast is thresholded at p < 0.05 FWE-corrected; at Session 3, the contrast is thresholded p < 0.005 uncorrected (> 10 voxels).

*Hc* Hippocampus, *Ent* Entorhinal cortex, *Phg* Parahippocampal gyrus.

In bold, regions tested with SVC; * regions significant after FWE correction at cluster level.

### Session 3

#### Memory performance

Four days after the first cued recall under metyrapone or placebo (i.e., when the active drug had been washed out), participants still remembered more emotional than neutral images when cued with the associated word in the fMRI scanner [main effect of emotion: F(1,22) = 5.152, *p* = 0.033, η^2^ = .190]. Planned follow-up comparisons indicated that lower memory recall following metyrapone compared to placebo condition was marginally evident for emotional photos [t(22) = -1.680, *p* = 0.053, r = 0.33; one-tailed] but not for neutral photos [t(22) = 0.214], *p* = 0.416, r = 0.05; one-tailed. However, the treatment by emotion interaction did not reach significance [F(1,22) = 3.835, *p* = 0.063, η^2^ = 0.14; Fig. 2B: cued recall_3_]. To validate the recall of the associated photos, another cued-recall test with a written description of the associated photos was given outside the scanner. This confirmed that recall of emotional photos was impaired four days after metyrapone intake compared to placebo [t(22) = -2.527, *p* = 0.009, r = 0.47; one-tailed]; as in the scanner, this was not the case for neutral photos (*p* = 0.166; one-tailed; Fig. 2B: cued recall_4_).

#### Imaging results

In the second fMRI session at Session 3, four days after the first recall test (i.e., when metyrapone had been washed out), cued recall of emotional vs. neutral photos after placebo again engaged several brain regions, including right amygdala, right anterior insula, right putamen, and left anterior cingulate, with only the amygdala activation remaining significant after SVC and FEW correction for multiple comparisons (*p* = 0.005, > 10 voxels; see Table 1B; Fig. 3C). After metyrapone, distinct activations were observed in right posterior insula and left temporal cortex, but not in the amygdala or anterior cingulate, with no distinctive activation surviving correction for multiple comparisons (*p* = 0.005 uncorrected, > 10 voxels; see Table 1B; Fig. 3D). In the planned interaction contrast for emotional hits versus misses relative to neutral hits versus misses in the placebo versus metyrapone conditions, we found differential activation for left hippocampus, left medial prefrontal cortex (including left anterior cingulate), left temporal pole, left thalamus, and left superior frontal gyrus (*p* = 0.005 uncorrected, > 10 voxels; see Fig. 3D and Table S4). Only the distinct engagement of left hippocampus and medial prefrontal cortex remained significant after applying SVC and FEW correction for multiple comparisons.

## Discussion

The present study adds to previous findings showing that pharmacologically induced cortisol suppression upon retrieval can weaken memory, including for emotional material, both acutely and in a long-lasting manner [8, 9]. Crucially, we also demonstrate here, in the placebo condition, that cued recall of emotional memories during the morning rise of cortisol is associated with activating a distinct brain network, including amygdala, hippocampus, and ventromedial prefrontal cortex. In contrast, cued recall of emotional memories under acute cortisol suppression does not engage these regions. Furthermore, cued recall performed four days later, when metyrapone was no longer effective (i.e., when metyrapone had been washed out and thus cortisol levels were no longer suppressed), still evoked distinct brain activation patterns when recalling emotional photos in conditions with prior administration of metyrapone or placebo. Thus, cortisol suppression persistently changes emotional memory representations in the brain.

Metyrapone administration robustly suppressed the subsequent morning cortisol rise and impaired performance in the cued recall task conducted in the fMRI scanner under this acute cortisol suppression state (Session 2), in accordance with previous studies [7–9]. Of note, despite the large effect size, the observed reduction in memory performance was numerically small and was not verified with a non-subjective memory measure and should therefore be interpreted with caution. Remarkably, cued recall of emotional vs. neutral photos in the fMRI scanner during Session 2 engaged a specific neural network associated with emotional processing and memory, including ventromedial prefrontal cortex, amygdala, and hippocampus. In previous studies, these brain regions have been associated with enhanced retrieval of emotional memories using recognition tasks and emotional context paradigms [5, 29, 30]. Most critically, the metyrapone-induced cortisol suppression in our study abolished the engagement of this emotion-associated brain network. This finding confirms our hypothesis derived from animal research [19, 31, 32], as suppressed cortisol levels do not allow for an emotion-driven increase in the activation of the amygdala, hippocampus, and prefrontal cortex, where glucocorticoid receptors are densely expressed [3, 33]. Moreover, contrasting emotional hits vs. misses to neutral hits vs. misses for placebo vs. metyrapone condition (triple interaction) directly unveiled a striking and selective difference in the activation of key memory-related regions, including amygdala, hippocampus, and parahippocampal regions. Altogether, this study indicates that cortisol suppression effects on emotional memory recall in humans are probably due to insufficient engagement of brain regions crucial for the enhanced retrieval of emotional memories, such as the ventromedial prefrontal cortex, amygdala, and hippocampus.

Four days after drug administration (Session 3), another cued recall in the fMRI scanner showed marginally lower memory performance for emotional photos in the metyrapone condition compared to the placebo condition. Impaired delayed memory performance for emotional photos after metyrapone was also found in our follow-up cued recall test which was tested with written descriptions of the photos after the fMRI session. These findings align with and extend previous data suggesting that metyrapone and associated cortisol suppression can impair the free recall of memories both acutely and in a long-lasting way [8, 9]. Interestingly, cued recall of emotional vs. neutral photos in the fMRI scanner four days after drug administration (Session 3) also showed distinct neural responses for memories previously retrieved in the metyrapone and placebo conditions. Emotional memories previously retrieved in the placebo condition were associated with enhanced activation of amygdala, anterior insula, putamen, and anterior cingulate, with only amygdala activation remaining significant after SVC and FWE correction. In contrast, emotional memories retrieved four days earlier in the metyrapone condition were now linked to enhanced activation of posterior insula, left transverse gyrus, and middle temporal gyrus, but not of amygdala or anterior cingulate; with no distinctive activation surviving correction. In addition, contrasting hits vs. misses for emotional vs. neutral photos in the placebo vs. metyrapone condition revealed a distinctive engagement of hippocampus, medial prefrontal cortex (including anterior cingulate), temporal pole, and superior frontal gyrus, with the activation of left hippocampus and medial prefrontal cortex surviving SFC and FEW correction, and, only a subregion of the medial prefrontal cortex coinciding with any activation in Session 2. This finding agrees with studies investigating system-level consolidation over time and a mnemonic processing shift from hippocampal to neo-cortical representations [34, 35]. Potential mechanisms for the observed differences in the neural correlates of emotional memories at Session 3 may reflect disrupted reconsolidation processing following memory reactivation [15, 36, 37], and/or reduced rehearsal-dependent strengthening of the memory trace as a consequence of the impaired memory performance in the metyrapone condition at Session 2 [38, 39]. In sum, our study shows that cortisol suppression upon the retrieval of emotional memories may alter neural memory representations for emotional events in a long-lasting way.

Understanding how emotional memories change is crucial for addressing intrusive memories in post-traumatic stress disorder (PTSD). Targeted treatments can effectively tackle memory retrieval, unlike the initial formation of traumatic memories. Prior studies indicate that reactivating emotional memories, along with cortisol or beta-adrenergic antagonist propranolol, can reduce the intensity of such memories [37, 40–44]. However, findings across studies have been inconsistent, with reports of enhancing, impairing, or no effects of hormonal modulation on reconsolidation [9, 45–48]. The present study examined emotional memory retrieval using the cortisol synthesis blocker metyrapone, and confirmed a subsequent impairment in emotional memory recall, converging with previous findings [7–9]. Moreover, our results reveal that impairments in emotional memory are linked to a disengagement of the neural network required for retrieving these memories. This deficit persists over time, indicating that emotional memories were associated with distinct neural representations in the metyrapone condition, compared to the placebo condition, even four days after treatment. Thus, cortisol suppression during the retrieval of emotional memories may represent a valuable strategy for attenuating emotional memories when they are distressing, highlighting its potential clinical relevance. More studies are needed to elucidate this perspective, considering the limitations of the present study and with the aim to re-test and extend its findings.

Nevertheless, it should be noted, that the discussed findings are observed under and after metyrapone administration, which on the top of inhibiting cortisol synthesis and therefore reducing cortisol levels, exerts additional effects on an organism. In particular, pharmacological decrease of cortisol disrupts negative feedback at the hypothalamic-pituitary-adrenal axis (HPA axis), which leads to an increase of the levels of other hormones, such as CRH and ACTH, with potential effects on cognitive functions [9, 49–51]. The possible consequences of this broader neuroendocrinological modulation of metyrapone action to the brain highlights the need for future studies, to investigate and clarify alternative or supplementary mechanisms for the observed effects.

Another limitation of the present study is that there are no measures of the basal cortisol levels at Session 1 and Session 3, despite evidence from previous studies that diurnal cortisol activity is comparable across different days for the same individuals [52, 53].

In conclusion, our study elucidates that cortisol suppression not only hinders the retrieval of emotional memories when treatment is effective but also impacts the later recall of these memories. This observed memory retrieval impairment under insufficient cortisol levels was associated with reduced engagement of brain regions necessary for emotional memory retrieval, such as amygdala, hippocampus, and prefrontal cortex. Furthermore, the neural correlates of recalling these emotional memories four days later remain distinctly altered in the human brain, suggesting that cortisol suppression can change neural memory representations for emotional events in a long-lasting way.

## Supplementary information

Supplemental Material

## Data Availability

The datasets generated and/or analysed during the current study are not publicly available because participant consent did not include provisions for public data sharing and MRI data are potentially identifiable and subject to data protection regulations, including the General Data Protection Regulation (GDPR). The data are available from the corresponding author on reasonable request, subject to applicable ethical and legal restrictions.
