# Supplementary material for: Cortisol suppression impairs retrieval of emotional memory and changes its neural correlates immediately and four days post-treatment
Source: Neuropsychopharmacology. 2026 Jul 23;51(11):1991–8. doi: 10.1038/s41386-026-02505-z (PMC13598141; doi:10.1038/s41386-026-02505-z)
Supplement: Supplementary file 1 [file 41386_2026_2505_MOESM1_ESM.docx]

**Supplemental Material**

Cortisol suppression impairs retrieval of emotional memory and changes its neural correlates immediately and four days post-treatment

Despina Antypa, Patrik Vuilleumier, Ulrike Rimmele

**Psychological control variables**

**Results**

**Session 1.** There were no differences between conditions in reported mood, positive/negative affect, experienced emotion, or measures of attention and working memory at Session 1 (all p > .130).

**Session 2.** There were no differences between conditions in reported mood, positive/negative affect, experienced emotion, and working memory at Session 2 (all p > .132). Metyrapone decreased performance in the attention d2 letter cancellation task at Session 2 (correctly canceled letters minus false alarms: M_M_ = 218.23, SE = 42.57 vs. M_PL_ = 319.27, SE = 37.68; t(21) = -3.213, p = .004, r = .57). Nevertheless, this effect on attention did not correlate with memory performance on that day (r = .189, p = .400), and it was not a significant covariate when added to the repeated measures model of memory performance (p = .670). Moreover, participants reported an overall slightly lower quality of sleep in the first morning after giving metyrapone (M_M_ = 2.96, SE = .11 vs. M_PL_ = 3.18, SE = .12; t(18) = -1.950, p=.067, r=.41).

Metyrapone did not affect recall latencies (time between the cue-word onset and the report of vivid photo recall) during this session (all p > .222; mean event duration 3.99 ± 1.96 s).

**Session 3.** Participants were able to correctly identify whether they had been administered metyrapone or placebo when asked to indicate what they thought they had been administered in a questionnaire given at the end of Session 3 of each condition (χ^2^(1) = 19.714, p < .001).

Metyrapone administration four days before did not affect recall latencies in Session 3 [time between cue-word onset and vivid recall report, all p > .198; mean event duration 4 ± 2.63 sec].

**Materials and Methods**

In both conditions, we assessed working memory, attention, mood, alertness, calmness, positive and negative affect, as well as experienced emotion at different time points during the three sessions (see Supplementary Table 6 for a detailed description). Working memory was evaluated using the Digit Span subtest (both forward and backward) of the Wechsler Adult Intelligence Scale (1); attention was measured with the d2 letter cancellation test (2); mood, alertness, and calmness were assessed using the Multidimensional Mood Questionnaire (3); positive and negative affect were measured with the Positive and Negative Affect Scale (PANAS) (4); and experienced emotion was evaluated using the original Self-Assessment Manikin scale (5).

Additionally, participants assessed their subjective sleep quality each night in the lab using the St. Mary’s Hospital Sleep Questionnaire (6). At the end of Session 3, for both conditions, they completed a questionnaire about which substance (metyrapone or placebo) they believed they had received.

**Imaging data acquisition and analysis**

**Materials and Methods**

The fMRI data were in a 3T whole-body MRI scanner (Trio TIM, Siemens, Germany) with a 32-channel head coil, using a fast-multiplexed echo-planar imaging (EPI) sequence optimized for blood-oxygen-level-dependent contrast and high temporal resolution, with the following parameters: TR = 650 ms, TE = 30 ms, flip angle = 50°, 36 interleaved slices, 64 x 64 pixels, isotropic voxel size of 3 mm³. The multiband acceleration factor was 4, and no parallel acquisition technique (PAT) was used. Structural images were acquired using a T1-weighted 3D sequence (MPRAGE, TR = 1900 ms, TE = 2.27 ms, TI = 900 ms, flip angle = 9°, 192 sagittal slices, voxel dimensions = 1 mm³ isotropic, 256 x 256 pixels, PAT factor = 2).

The Statistical Parametric Mapping software SPM8 (Welcome Trust Centre for Neuroimaging, London, UK) (see Supporting Information for more details). was used to preprocess and analyze the EPI images with standard procedure (see Supporting Information for more details). The preprocessing of functional images included spatial realignment to the mean of all images, co-registration to the anatomical scan, spatial normalization to the standard Montreal Neurological Institute (MNI) EPI template, and spatial smoothing with an isotropic 8 mm full-width at half-maximum Gaussian kernel.

The 12 event types included into the single general linear model (GLM) for each session were: NHits_M, NMisses_M, EHits_M, EMisses_M, NRest_M, ERest_M, NHits_PL, NMisses_PL, EHits_PL, EMisses_PL, NRest_PL, ERest_PL; N: Neutral, E: Emotional, M: Metyrapone, PL: Placebo). Hits were defined as the trials in which participants reported recalling the photo previously paired with the presented cue word. Misses were the trials in which participants reported no memory of the photo paired with the cue word or did not respond. For both Hits and Misses, the event duration was measured from the onset of the cue word to the participants’ response. This includes the moment they indicated that they vividly recollected each photo for "Hits" and the moment they said they could not recollect the photo for "Misses." It also encompasses the end of the trial when no response was given (7,8). Using this variable response-based duration design, the hemodynamic response was then modeled for each covariate of interest, including response time for each trial. Rest runs after emotional and neutral blocks were included in the first-level models for sessions 2 and 3 (onset set as the beginning of the rest, and duration as a fixed interval of rest, i.e., 180 sec as event duration), but they were not used in the second-level analyses. High-pass temporal filtering was applied using a 128-s cut-off.

Supplemental Figures

**Fig. S1.** Assessment of psychological control variables (in bold) across the experiment sessions.

**Fig. S2.** Parameter estimate plots extracted for left medial prefrontal and right amygdala for individual trial types in the two conditions, for Sessions 2 and 3.

(A) Parameter estimate plots extracted for left medial prefrontal cortex (-3 41 -17) for emotional and neutral hits in placebo and metyrapone condition at Session 2.

(B) Parameter estimate plots extracted for right amygdala (27 2 -29) for emotional and neutral hits in placebo and metyrapone condition at Session 2.

(C) Parameter estimate plots extracted for left medial prefrontal cortex (-3 41 -17) for emotional and neutral hits in placebo and metyrapone condition at Session 3.

(D) Parameter estimate plots extracted for right amygdala (27 2 -29) for emotional and neutral hits in placebo and metyrapone condition at Session 3.

Supplemental Tables

**Table S1.** Output of linear mixed model on cortisol levels with fixed effects of factors treatment (placebo/metyrapone), time (10-time points of the saliva samples/condition), and random effects of the factor subject.

| Intercept | Estimate | SE | tStat | pValue | 95 % CI  (Lower, Upper) |
| --- | --- | --- | --- | --- | --- |
| Placebo at 3:45 am  (Baseline for PL) | .063 | .062 | 1.011 | .312 | -.059, .185 |
| Placebo at 6:45 am | .494 | .081 | 6.164 | <.001 | .336, .651 |
| Placebo at 7:00 am | .665 | .079 | 8.466 | <.001 | .510, .819 |
| Placebo at 7:15 am | .857 | .079 | 10.914 | <.001 | .702, 1.011 |
| Placebo at 8:30 am | .697 | .079 | 8.796 | <.001 | .542, .854 |
| Placebo at 8:45 am | .707 | .081 | 8.708 | <.001 | .548, .867 |
| Placebo at 9:00 am | .637 | .079 | 8.041 | <.001 | .482, .793 |
| Placebo at 9:15 am | .594 | .081 | 7.308 | <.001 | .434, .753 |
| Placebo at 9:30 am | .569 | .080 | 7.094 | <.001 | .411, .726 |
| Placebo at 9:45 am | .563 | .082 | 6.847 | <.001 | .402, .725 |
| Metyrapone at 3:45 am  (Baseline for M) | -.056 | .081 | -.687 | .492 | -.215, .104 |
| Metyrapone at 6:45 am | -.387 | .113 | -3.418 | <.001 | -.610, -.165 |
| Metyrapone at 7:00 am | -.859 | .113 | -7.624 | <.001 | -1.081, -.638 |
| Metyrapone at 7:15 am | -1.145 | .112 | -10.209 | <.001 | -1.365, -.925 |
| Metyrapone at 8:30 am | -1.121 | .116 | -9.668 | <.001 | -1.348, -.893 |
| Metyrapone at 8:45 am | -1.128 | .116 | -9.711 | <.001 | -1.356, -.899 |
| Metyrapone at 9:00 am | -1.172 | .113 | -10.404 | <.001 | -1.393, -.899 |
| Metyrapone at 9:15 am | -1.253 | .115 | -10.867 | <.001 | -1.479, -1.026 |
| Metyrapone at 9:30 am | -1.011 | .113 | -8.922 | <.001 | -1.233, -.788 |
| Metyrapone at 9:45 am | -.950 | .116 | -8.191 | <.001 | -1.178, -.722 |

tStat: *t* statistic; CI: confidence interval

Table S2. Session 2: Emotional hits vs. neutral hits in placebo vs. metyrapone condition.

| Region | Side | Number of Voxels | *Z* score | MNI coordinates (mm) |
| --- | --- | --- | --- | --- |
| Parietal operculum  Temporal pole  Subcallosal area  **Amygdala/Hc/Ent/Phg**  Temporal pole  Transverse temporal gyrus  **Medial frontal cortex**  Midcingulate cortex  Ventral diencephalon  Middle frontal gyrus  Postcentral gyrus  Postcentral gyrus  Lingual gyrus  Cerebellum  **Amygdala** | R  R  R  R  R  R  R  L  L  L  L  L  R  R  R  L  L | 32  42  22  115  26  25  70  26  11  18  22  19  17  11  10 | 3.16  3.05  3.05  3.00  2.95  2.89  3.00  2.95  2.94  2.92  2.90  2.87  2.80  2.80  2.76  2.73  2.70 | 39 -28 22  54 2 -11  9 20 -11  **27 2 -29***  **18 -4 -23***  **12 -19 -8***  42 -28 -5  -33 -31 16  **-3 44 -17***  15 -13 46  -6 -13 -17  -42 -50 -2  63 -16 40  33 -10 67  6 -70 -8  -3 -64 -38  **18 -4 -26*** |

Activations associated with the retrieval of emotional vs. neutral photos upon cue. Contrast thresholded at p < .005 uncorrected (> 10 voxels).

Hc: Hippocampus; Ent: Entorhinal cortex; Phg: Parahippocampal gyrus.

In bold, regions tested with SVC; ***** regions significant after FWE correction at cluster level.

Table S3. Session 2: Emotional (hits vs. misses) vs. neutral (hits vs. misses) in placebo vs. metyrapone condition.

| **Region** | **Side** | **Number of Voxels** | ***Z* score** | **MNI coordinates (mm)** |
| --- | --- | --- | --- | --- |
| Occipital pole  Thalamus  Lingual gyrus  Cerebellum  **Amygdala/Hc/Phg**  Cerebellum  Lingual gyrus  Cerebellum  Supramarginal gyrus  Thalamus  Superior parietal lobule  Middle frontal gyrus | R/L  R  L  R  R  R  R  R  R  L  L  L  L  R | 12  43  19  16  28  16  13  17  12  13  14  10 | 2.80  2.74  2.72  2.72  2.70  2.64  2.59  2.68  2.67  2.65  2.65  2.64  2.63  2.62 | 0 -103 10  0 -28 4  -9 -85 -17  18 -19 -32  **24 -25 -20***  **18 -16 -23***  **18 -4 -20***  42 -49 -29  3 -67 -2  -27 -61 -23  -54 -40 52  -24 -31 7  -24 -58 55  51 26 28 |

Activations associated with the retrieval of emotional vs. neutral photos, upon cue. Contrast thresholded at p < .005 uncorrected (> 10 voxels).

Hc: Hippocampus; Phg: Parahippocampal gyrus.

In bold, regions tested with SVC; ***** regions significant after FWE correction at cluster level.

Table S4. Session 3: Emotional (hits vs. misses) vs. neutral (hits vs. misses) in placebo vs. metyrapone condition.

| **Region** | **Side** | **Voxel** | ***Z* score** | **MNI coordinates (mm)** |
| --- | --- | --- | --- | --- |
| Superior frontal gyrus  Temporal pole  **Hc/Phg**  Thalamus  **Medial frontal cortex**  Middle temporal gyrus | L  L  L  L  L  L  L  L  L | 65  30  21  41  74  14 | 3.14  3.00  3.00  2.84  2.96  2.92  2.89  2.73  2.81 | -6 62 28  -42 20 -35  **-21 -19 -11***  **-12 -22 -23***  -3 -16 -2  **-3 56 -5***  **-6 44 -8***  **-6 41 1***  -57 -16 -26 |

Activations associated with the retrieval of emotional vs. neutral photos, upon cue. Contrast thresholded at p < .005 uncorrected (> 10 voxels).

Hc: Hippocampus; Phg: Parahippocampal gyrus.

In bold, regions tested with SVC; ***** regions significant after FWE correction at cluster level.

**SI References**

1. Weshler D (1981): *Wechsler Adult Intelligence Scale -Revised.* New York: Harcourt Brace Jovanovich.

2. Brickenkamp MM, Zillmer E (1998): *The D2 Test of Attention.* Seattle, Washington: Hogrefe & Huber Publishers.

3. Steyer R, Schwaenkmezger P, Notz P, Eid M (1997): *Der Mehrdimensionale Befindlichkeitsfragebogen (MDBF).* Goettingen: Hogrefe.

4. Watson D, Clark LA, Tellegen A (1988): Development and validation of brief measures of positive and negative affect: The PANAS scales. *Journal of Personality and Social Psychology* 54: 1063–1070.

5. Bradley MM, Lang PJ (1994): Measuring emotion: The self-assessment manikin and the semantic differential. *Journal of Behavior Therapy and Experimental Psychiatry* 25: 49–59.

6. Ellis BW, Johns MW, Lancaster R, Raptopoulos P, Angelopoulos N, Priest RG (1981): The St. Mary’s Hospital Sleep Questionnaire: A Study of Reliability. *Sleep* 4: 93–97.

7. Sharot T, Delgado MR, Phelps EA (2004): How emotion enhances the feeling of remembering. *Nat Neurosci* 7: 1376–1380.

8. Daselaar SM, Rice HJ, Greenberg DL, Cabeza R, LaBar KS, Rubin DC (2008): The Spatiotemporal Dynamics of Autobiographical Memory: Neural Correlates of Recall, Emotional Intensity, and Reliving. *Cerebral Cortex* 18: 217–229.
